# The endocannabinoid system: directing eating behavior and macronutrient metabolism

**DOI:** 10.3389/fpsyg.2014.01506

**Published:** 2015-01-06

**Authors:** Bruce A. Watkins, Jeffrey Kim

**Affiliations:** ^1^Department of Nutrition, University of CaliforniaDavis, Davis, CA, USA; ^2^Division of Cardiovascular Medicine, Department of Internal Medicine, University of CaliforniaDavis, Davis, CA, USA

**Keywords:** food intake behavior, endocannabinoids, polyunsaturated fatty acids, appetite, cannabinoid receptors, brain and neuronal function

## Abstract

For many years, the brain has been the primary focus for research on eating behavior. More recently, the discovery of the endocannabinoids (EC) and the endocannabinoid system (ECS), as well as the characterization of its actions on appetite and metabolism, has provided greater insight on the brain and food intake. The purpose of this review is to explain the actions of EC in the brain and other organs as well as their precursor polyunsaturated fatty acids (PUFA) that are converted to these endogenous ligands. The binding of the EC to the cannabinoid receptors in the brain stimulates food intake, and the ECS participates in systemic macronutrient metabolism where the gastrointestinal system, liver, muscle, and adipose are involved. The EC are biosynthesized from two distinct families of dietary PUFA, namely the n-6 and n-3. Based on their biochemistry, these PUFA are well known to exert considerable physiological and health-promoting actions. However, little is known about how these different families of PUFA compete as precursor ligands of cannabinoid receptors to stimulate appetite or perhaps down-regulate the ECS to amend food intake and prevent or control obesity. The goal of this review is to assess the current available research on ECS and food intake, suggest research that may improve the complications associated with obesity and diabetes by dietary PUFA intervention, and further reveal mechanisms to elucidate the relationships between substrate for EC synthesis, ligand actions on receptors, and the physiological consequences of the ECS. Dietary PUFA are lifestyle factors that could potentially curb eating behavior, which may translate to changes in macronutrient metabolism, systemically and in muscle, benefiting health overall.

## INTRODUCTION

Decades ago, it was recognized that macronutrient expenditure and maintenance of dietary intake are controlled by complex physiological processes involving endocrine, neurological, and behavioral factors (Mayer and Thomas, 1967). More recently, the ECS was found to be integral in the control of food intake and a target to mitigate obesity (Cota, 2007). The cannabinoid receptors of this system are present in large numbers in the brain, and their activation by endogenous agonists, called EC, has revealed yet another aspect of the neuroendocrine system’s participation in caloric intake (Mackie, 2008; Viveros et al., 2008). Furthermore, studies using pharmacological antagonists of the cannabis receptors in the brain have demonstrated marked improvements in weight loss and appetite control in humans but with undesirable side effects that include heart disease and depression (Di Marzo and Despres, 2009). The first discovered EC were found to be biosynthesized from PUFA of the n-6 family, however, those derived from the n-3 PUFA family have shown the potential of a dietary approach to modulate activation of the cannabinoid receptors to diminish food intake and downstream events that reduce macronutrient metabolism directed toward fat accumulation (Naughton et al., 2013). Indirect evidence to support this premise comes from studies in n-3 PUFA deficient-mice which abolished EC mediated neuronal functions (Lafourcade et al., 2011) and where feeding n-3 PUFA decreased the levels of the n-6 PUFA derived EC (Watanabe et al., 2003; Alvheim et al., 2012). The n-3 PUFA may lower activation of cannabinoid receptors, thereby attenuating food intake (Watkins et al., 2010; Kim et al., 2013).

The ECS includes both synaptic and peripheral signaling functions, and the cannabinoid receptors are found in many organs and tissues besides the nervous system (Mackie, 2008). Cannabinoid receptors are G protein-coupled receptors that upon activation, lead to multiple complex signaling pathways (Lafourcade et al., 2011). AEA and 2-AG, both derived from (20:4n-6 an n-6 PUFA), are the two most abundant endogenous EC for the cannabinoid receptors CB1 and CB2 (Bisogno et al., 1999; Bosier et al., 2010). However, other EC have been identified which include those biosynthesized from n-3 PUFA, such as eicosapentaenoyl ethanolamide from eicosapentaenoic acid (EPA or 20:5n-3) and docosahexaenoyl ethanolamide from docosahexaenoic acid (DHA or 22:6n-3; Rossmeisl et al., 2012; Kim et al., 2014a). Binding affinities differ between the various EC ligands for the cannabinoid receptors, and as such, result in varying degrees of activation of the receptor and downstream effects (Mackie, 2008), which, in the case of the central nervous system (CNS), includes blocking of neurotransmitter release from the presynaptic neuron (Okamoto et al., 2004). The binding affinities of the EC derived from either EPA or DHA toward cannabinoid receptors are shown to be comparatively weaker than the AA-derived EC (Brown et al., 2010). In cells and tissues, a dynamic competition exists in the biochemistry of the PUFA families, and recently, their actions on ECS gene expression in both humans and rodents have varied (Watkins et al., 2010; Kim et al., 2013). Examples of how PUFA and EC change the expression of ECS-related genes have been reported in C2C12 myoblast cultures (Kim et al., 2014a) and in mice (Hutchins-Wiese et al., 2012). In studies with rodents, the consumption of dietary lipids varying in the amounts of n-6 and n-3 PUFA as well as monounsaturated fatty acids is now believed to be one approach to control appetite and obesity by changing endogenous levels of EC and subsequent receptor activation (Naughton et al., 2013).

During development, measurable increases in the expression of cannabinoid receptors (mRNA and protein) and circulating levels of EC have been reported, along with receptor binding in the fetal and early post-natal brains of rodents (Berrendero et al., 1999). Along with aging, it is suggested that a decrease in ECS activity is associated with a decline in neuroprotective regulation in the brain of rodents (Bilkei-Gorzo, 2012). Evidence supporting the neuroprotective benefits conferred by ECS intervention is scarce but growing (Goncalves et al., 2008; Marchalant et al., 2008, 2009). While the means of protection are a result of pharmacological intervention on the cannabinoid receptors, aging and environmental factors, such as dietary n-6 PUFA, may provide a protective mechanism to maintain circulating levels of EC that would offset the decline of biosynthetic enzyme levels for EC synthesis. However, at the same time, the n-3 PUFA may help to minimize overstimulation of the ECS during obesity to reduce the n-6 PUFA derived EC (Kim et al., 2014b). Thus, dietary PUFA studies are of great research interest to better understand eating behavior and excess caloric intake in the adolescent and young adult populations, as well as suppressed appetites in older adults. The aim of this research is to determine how specific families of dietary PUFA alter EC levels, receptor excitability, and signaling to improve health outcomes throughout the life cycle.

Herein, we describe the ECS and how it is integrated in the biological control of food intake; maintenance of the ECS; actions of PUFA on genes of the ECS; relationships between muscle, adipose, and liver; and aging. Throughout this review, we will suggest research to further elucidate the interactions between PUFA and the ECS and recommend approaches to advance the understanding of how EC and receptors function in the body.

## OVERVIEW OF THE ENDOCANNABINOID SYSTEM IN THE BRAIN AND PERIPHERAL ORGANS

The functional components of the ECS are the cannabinoid receptors, biosynthesis and degradation enzymes of the EC ligands, and their signaling pathways (Murray et al., 2007). Although the brain and CNS exhibit all of these attributes of the ECS (Mackie, 2008), the components of this system have also been shown to be present and active in several organs, tissues, and cells (Vettor et al., 2008; De Petrocellis and Di Marzo, 2009). In the late 1980s the central receptor for the psychotropic component of cannabis, tetrahydrocannabinol, was identified as cannabinoid receptor 1 (CB1; Devane et al., 1988), and it was later cloned in 1990 by Matsuda et al. (1990). Endogenous agonists for CB1 and the predominantly localized peripheral cannabinoid receptor, CB2, were also identified in the early and mid-1990s (Mechoulam et al., 1995). AEA was first discovered as a cannabinoid ligand, followed by 2-AG; these fatty acid derived compounds are made on demand and act locally in a paracrine or autocrine fashion. CB1 has since been identified to be localized on numerous peripheral tissues such as adipose, liver, and muscle (Kim et al., 2013). CB2 was first identified in immunocompetent cells and has since been identified in various peripheral organs (i.e., bone, muscle, heart) and the CNS, albeit to a lesser degree than CB1 expression. The cannabinoid receptors are Gi/o protein-coupled receptors and share 44% overall identity (Howlett, 2005). Upon activation, a signaling cascade occurs and results in the inhibition of adenylyl cyclase, cAMP, and protein kinase A. The signaling pathways of the ECS are recognized as a target for therapeutic applications (Bosier et al., 2010), but the complex relationships between the endogenous ligands, receptors, and signaling are poorly understood.

The ECS signaling pathways are multifaceted because the many endogenous ligands, including those derived from the n-6 and n-3 PUFA families, result in varying degrees of receptor activation and involve different intracellular transduction pathways to influence numerous physiological functions (Bosier et al., 2010). The modification of receptor activation by the different endogenous and exogenous ligands selectively directs the downstream actions of the ECS *in vivo*. At present, the actions of the multitude of ligands on the ECS are not well described in the literature. Potentially, modification of the ECS by endogenous ligands derived from dietary PUFA may be a means to blunt food intake and alter physiological processes to reduce obesity and improve skeletal muscle response to glucose and insulin sensitivity (Kim et al., 2013, 2014a). These relationships are now under investigation in rodents and humans (Viveros et al., 2008); however, there are few clinical published studies (McPartland et al., 2014). Adequate amounts of dietary n-3 PUFA are necessary for proper cannabinoid signaling, and the long chain n-3 PUFA, both DHA and EPA, moderate the cellular levels of AEA and 2-AG, thus acting as a means to alter ECS signaling to reduce over stimulation of signaling and its negative consequences on health, such as obesity (Kim et al., 2013; McPartland et al., 2014).

Additional downstream signaling pathways resulting from the ECS include extracellular receptor kinase, mitogen activated protein kinase, c-Jun *N*-terminal kinases, and c-fos (Wartmann et al., 1995; Howlett, 2005). CB1 activation in neurons and other cranial tissues is responsible for inhibition of intracellular cAMP, dephosphorylation of potassium and calcium ion channels, and increased intracellular free Ca^2+^ (Howlett, 2005). CB2 activation in immune cells is generally considered anti-inflammatory and immunomodulatory (Han et al., 2009; Hao et al., 2010). AEA acts as a partial CB1 agonist and a weak CB2 agonist, while 2-AG is a full CB1 and CB2 agonist (Bisogno et al., 2005). Two additional receptors, GPR55 (Ryberg et al., 2007) and transient receptor potential vanilloid type 1 (Ross, 2003) are also activated by AEA; although, their interactions with the ECS are currently under investigation.

The next research area for the ECS must include characterization of the downstream actions and should also include the relationships of related substrates, such as the ligands for the EC receptors and those that can be used by cyclooxygenase (Kim and Watkins, 2014). This research will have implications on brain functions, inflammatory status, and disease pathogenesis. The physiological aspects of this research have implications in obesity and insulin resistance, but with regards to food intake, such research can integrate the role of the ECS in systemic macronutrient metabolism and fat accretion. The ECS has some impact on directing systemic macronutrient metabolism, which involves the intestinal tract, liver, muscle, and adipose (Viveros et al., 2008; Kim et al., 2013). Hence, the interest in the ECS on food intake and energy balance is of vital importance to human health (Di Marzo and Matias, 2005; Tibirica, 2010).

## ENDOCANNABINOID BIOSYNTHESIS, DEGRADATION AND THE BRAIN

Arachidonylethanolamide and 2-AG are biosynthesized from AA in the phospholipid membrane and are the two most studied endogenous ligands for the cannabinoid receptors. AEA is synthesized from AA in the sn-1 position on phospholipids and is found at pmol/g concentrations in tissues, whereas 2-AG is formed from AA in the sn-2 position. AA is more abundant in the sn-2 position of the phospholipids in the cell membrane, leading to higher tissue concentrations of 2-AG (ng/g range) than AEA (Bab et al., 2009). Currently, there are three known routes of AEA formation from AA. The two step synthesis by NAPE-PLD is the major pathway, where AA is first cleaved as a phosphatidylethanolamine by an acyltransferase to form NAPE and then the NAE is released from NAPE by a selective PLD (Okamoto et al., 2004). When AA is the starting substrate, the released NAE is AEA. NAE has been previously found to function as a signaling molecule in various tissues, primarily in the CNS (Schmid, 2000). Another route of synthesis is via hydrolysis of NAPE from phospholipase C, forming an *N*-arachidonoylethanolamine phosphate which can then be hydrolyzed by a phosphatase to form AEA. Synthesis of AEA can also be achieved by a condensation pathway where a FAAH works in reverse, starting with AA and ethanolamine. As for the synthesis of 2-AG, various pathways have been characterized (Sugiura et al., 1995). For example, phosphatidylinositol (PI) can be hydrolyzed by phospholipase A, forming a lysophosphatidylinositol which is then hydrolyzed by phospholipase C to form 2-AG. Additionally, 2-AG can also be produced in response to a stimulus, such as ionomycin, in which PI is hydrolyzed by phospholipase C forming triacylglycerol (DAG) and hydrolyzed once more by DAG lipase (DAGL). A third mechanism in forming 2-AG is via conversion of 2-arachidonoyl lysophosphatidic acid to 2-AG, which is accomplished by monoacylglycerol kinase. Degradation of AEA, and to a lesser degree 2-AG, occurs by FAAH to produce AA and an ethanolamide. The degradation of 2-AG occurs by MAGL to form AA and monoglycerol (Sugiura et al., 2002).

Previously, NAPE-PLD was shown to be expressed by particular populations of neurons in the brain, specifically targeting axon and axon terminals to mediate anterograde signaling at synapses (Egertova et al., 2008). This is evident in the observations where AEA acts as a retrograde messenger to bind to the axonal terminal of presynaptic neurons in the hippocampus (Wilson and Nicoll, 2001; Alger, 2002).

Agonists and inverse agonist actions on the cannabinoid receptors result in high to low responses, compared to basal cellular levels (Mackie, 2008). The *in vitro* response is dependent upon the binding affinity of the ligand to the cannabinoid receptor, the activation of the receptor, and the resulting downstream actions through the signaling pathways, which include gene expression; however, these are not well characterized *in vivo* (Bosier et al., 2010). What is clear is that the ECS in the brain controls food intake via the hypothalamus and limbic systems, where activation of cannabinoid receptors induces fat accumulation (Viveros et al., 2008). In addition, activation of receptors may divert macronutrient metabolism toward lipid synthesis in adipose (Osei-Hyiaman et al., 2005; Bluher et al., 2006). Moreover, dietary PUFA directly affect the concentration of specific EC in blood and tissues *in vivo* (Artmann et al., 2008; Wood et al., 2010; Kim et al., 2014b). The effects of dietary n-6 PUFA on the enzymes of synthesis and degradation of the EC is worthy of investigation *in vivo* and in cell cultures (Kim et al., 2014a). Conversely, sufficient evidence supports the need to conduct human studies that examine the full extent in which n-3 PUFA derived EC alter the ECS in health and in controlling obesity and insulin resistance (McPartland et al., 2014).

## ENDOCANNABINOIDS, THE GASTROINTESTINAL TRACT, AND CONTROL OF FOOD INTAKE

The ECS is well known to be involved in the regulation of appetite, food intake, and energy metabolism (Tibirica, 2010; Kim et al., 2011). Initially, it was thought that the effects of EC were localized in the CNS. The EC can act as neurotransmitters between neurons in various regions of the brain (Bermudez-Silva et al., 2012). They behave as retrograde messengers on CB1 at presynaptic glutamatergic terminals in the hypothalamus, resulting in the inhibition of the release of the excitatory neurotransmitter glutamate and leading to an overall suppressive effect on neuroendocrine function (Di et al., 2003). One of the major consequences of ECS action on the hypothalamus is affecting neuroendocrine functioning (Harmon and Aliapoulios, 1972). From an evolutionarily perspective, the primary physiological function of the ECS appears to shift energy balance toward energy storage (Piazza et al., 2007), and thus, can lead to fat accumulation. While the necessity for the ECS was more obvious for our hunter and gatherer ancestors when food supply was not guaranteed, the technological advances of today provide greater stability in regards to food availability. However, today this is more of a disadvantage with regard to being overweight and obese. An excessive food supply and an overactivation of CB1 can lead to overeating and a susceptibility of metabolism to favor energy storage and obesity (Piazza et al., 2007).

Stimulation in the CNS of CB1 by AEA and 2-AG increases hyperphagia, and the response is a higher food intake (Williams and Kirkham, 1999; Kirkham et al., 2002). The ECS controls food intake in two ways: (1) reinforces the motivation to find and consume food with high incentive value, and (2) induces appetite by regulating levels and actions of orexigenic and anorectic mediators (Di Marzo and Matias, 2005; Monteleone et al., 2005). The hyperphagic properties of EC were reported in Williams and Kirkham (1999) when AEA was injected peripherally to stimulate feeding behavior and overeating in satiated rats, an effect that was attenuated by selectively blocking CB1 with the antagonist SR141716. AEA caused a modest hyperphagic response that appeared over a longer time course, as compared to Δ^9^-THC (Williams and Kirkham, 1999). Injection of 2-AG into the nucleus accumbens shell, an area of the basal forebrain associated with appetite stimulation, induced eating in rats (Kirkham et al., 2002). This increase in food intake from 2-AG treatment was prevented by pre-treatment with the antagonist SR141716, while the CB2 antagonist SR144258 had no effect; thus, it was demonstrated that the hyperphagic properties of AEA and 2-AG are specifically mediated by central CB1 (Williams and Kirkham, 1999; Kirkham et al., 2002). Besides reinforcing the action of SR14176 on suppressing food intake in the rat, behavioral aspects related to motivational processes in both the appetitive and consummatory phases of feeding behavior are involved (Thornton-Jones et al., 2005). Subsequently, others reported that the CB1 receptor antagonists/inverse agonists (e.g., Rimonabant, analog AM 251) actions on reducing food intake appears to be linked to or mediated by behavioral aspects in the rat (Tallett et al., 2007). Adiponectin mRNA and plasma adiponectin were elevated in vehicle-treated chow-fed animals compared to obese controls, and did not differ between rimonabant-treated and pair-fed animals. The similarities between rimonabant-treated and pair-fed animals in body weight loss and the absence of differences in measures of adiponectin activity between drug-treated and pair-fed animals suggest that the outcomes of this experiment were solely mediated by the drug-induced reduction in food intake (Thornton-Jones et al., 2006). Others have reported a reduced but transient food intake in rats treated with a neutral CB1 receptor antagonist AM4113 (Cluny et al., 2011). Weight control resulting in a lower food intake with AM4113 was also observed in the rats that were subjected to pair-feeding. With regard to sex differences and diet, Foltin and Haney (2007) found that baboon males ate more food pellets than females, few other sex differences were observed in this study.

Signals to the brain from the small intestines and other organs of the gastrointestinal system play a role in regulating energy balance, and the ECS appears to have a role in these pathways (Di Marzo and Matias, 2005). The interaction between the gastrointestinal tract and the ECS is carried out by both endocrine and neural pathways. CB1 is present in neurons of the enteric nervous system and in sensory terminals of vagal and spinal neurons in the gastrointestinal tract (Massa et al., 2005). Activation of CB1 is shown to modulate nutrient processing, such as gastric secretion, gastric emptying, and intestinal motility. Intestinal derived hormones such as CCK and the adipocyte derived hormone, leptin, decrease food intake while ghrelin has the opposite effect by increasing appetite (Pagotto et al., 2006). These hormones function as satiety and hunger signals by triggering nerve impulses in sensory nerves that travel to the hindbrain and hypothalamus via blood. Food intake and hunger may be under the influence of the ECS by regulating the expression and action of orexigenic and anorectic mediators that originated from the hypothalamus. Leptin has also been found to lower 2-AG and AEA levels in the hypothalamic region in rats (Di Marzo et al., 2001). The higher levels of EC in ob/ob and db/db mice support this observation. Remarkably, it has been reported that inactivation of CB1 results in a decrease of plasma insulin and leptin levels (Ravinet Trillou et al., 2004). CB1 is shown to co-localize with the food intake inhibiting neuropeptide, corticotrophin-releasing hormone, in the paraventricular nucleus of the hypothalamus, and with the two orexigenic peptides, melanin-concentrating hormone in the lateral hypothalamus and with pre-pro-orexin in the ventromedial hypothalamus (Inui, 1999; Horvath, 2003). CB1 knockout (KO) mice showed higher levels of CRH mRNA, suggesting that hypothalamic EC receptors are involved in energy balance and may be able to mediate food intake (Cota et al., 2003).

The gastrointestinal tract is a site for EC production. It has been reported that feeding influences levels of EC, such as AEA. Gomez et al. (2002) found that after a 24-h fast, AEA levels in the small intestine were seven times higher than that of a littermate that had not fasted. When the ECS was blocked via antagonist (SR141716), food intake was reduced in fasted and partially satiated rats. Thus this work suggests that EC levels are responsive to nutrient status. By increasing n-3 PUFA intake, the dietary ratio of n-6/n-3 PUFA would be lowered and lead to a decrease in the synthesis of AEA and 2-AG from AA. This would mimic the hormonal and behavioral alterations that are observed in animals with treatment of a CB1 antagonist. This association between PUFA and EC supports the premise that a lower dietary ratio of n-6/n-3 PUFA would lead to a reduction in food intake by dietary manipulation of tissue AA levels.

Another route where the ECS has been found to regulate food intake is through the vagus nerve, which connects the medulla and brainstem nuclei associated with satiety with the gastrointestinal tract to monitor the status of digestive processes. After consuming food, CCK is secreted from the duodenum and then binds to CCK receptors that are located on afferent terminals of the vagus nerve. The signal is taken up the vagal axon and to the hypothalamus to signal the decrease of food intake. Leptin receptors have also been found on these same nerve terminals. Reports have indicated decreased CB1 receptor mRNA in rats that had been fed after previously fasting or receiving CCK. In addition, in a study looking at the effect of leptin, the investigators reported that acute leptin treatments reduced AEA in the hypothalamus (Di Marzo et al., 2001). The hypothalamus plays a critical role in receiving signals from peripheral organs to inform the brain of the state of energy status (Kirkham et al., 2002). Upon eating, wild-type rats were observed to have reduced hypothalamic 2-AG levels compared to when they were previously fasted.

An increase in food intake was observed when ghrelin was infused into the paraventricular nucleus of the hypothalamus in rats (Tucci et al., 2004). However, when a CB1 receptor antagonist was added to the ghrelin treated animal, the food intake returned to baseline. Elevated levels of the endogenous ligands, AEA and 2-AG, have also been found in obese individuals (Engeli et al., 2005; Osei-Hyiaman et al., 2005) and correlates with intra-abdominal adiposity (Cote et al., 2007). Additionally, diet-induced obese mice demonstrated higher levels of AEA and 2-AG in hippocampal regions and displayed an increase in DAGL, which is one of the enzymes responsible for the synthesis of 2-AG (Massa et al., 2010). In the same study, ECS-mediated synaptic plasticity was observed to have changed in the CA1 region, as depolarization-induced suppression of inhibition and long-term depression of inhibitory synapses were enhanced. This finding demonstrated the potential for the ECS to remodel and influence aspects of cognition. In another study, AEA injection into the ventromedial hypothalamus of satiated rats induced significant appetite stimulation through CB1 receptor activation (Jamshidi and Taylor, 2001).

Although feeding a mixture of different n-3 PUFA was found to increase plasma leptin in blood of insulin-resistant rats, food intake did not change (Peyron-Caso et al., 2002). While leptin functions to reduce appetite and increase energy expenditure, resistance to leptin’s effects is well documented in metabolic complications, such as obesity (Myers et al., 2010, 2012). Recently, treatment with a CB1 inverse agonist was shown to reverse leptin resistance and reduce obesity in diet induced obese mice (Tam et al., 2012). In another study, both plasma insulin and leptin levels were lower in CB1 KO mice, compared to wild-type mice (Ravinet Trillou et al., 2004). Both exogenous cannabinoids and AA-derived EC increase food intake and promote weight gain via CB1 receptor activation (Jamshidi and Taylor, 2001; Kirkham et al., 2002). From these studies, one can surmise that an overactivation of the ECS is indicative of the increased adiposity observed with obesity. In addition, leptin status appears to be influenced by the levels of endogenous cannabinoids, thus validating the idea that the ECS plays a major role in energy homeostasis. Unfortunately, the precise actions of the n-6 and n-3 families of dietary PUFA on activation of cannabinoid receptors and their signaling downstream is not known. For this reason, it is necessary to investigate how dietary PUFA influence the ECS in order to understand food intake and macronutrient metabolism and their effects on fat accretion and insulin sensitivity.

## DIETARY PUFA AND THE ENDOCANNABINOIDS

It is well recognized that dietary PUFA can alter the fatty acid composition of glycerolipids of cells, tissues, and organs in the body with some significant physiological outcomes (Watkins et al., 2006; Li et al., 2010; Hutchins-Wiese et al., 2012). In addition, the remodeling of plasma membrane PUFA composition is well documented in humans. Moreover, families of n-3 PUFA change membrane phospholipid composition in most organs, including the brain. Feeding the long chain n-3 PUFA, such as EPA and DHA, will increase their concentrations *in vivo*, and to some extent will lower the concentrations of n-6 PUFA, specifically AA. Thus, remodeling the phospholipid composition of cell membranes and organelles by dietary PUFA is a means to change substrate for and the biosynthesis of prostanoids (Watkins et al., 2000), and more recently for the biosynthesis of EC (Watkins et al., 2010; Kim and Watkins, 2014; Kim et al., 2014a).

As suggested, tissue EC levels are responsive to substrate PUFA availability and can be modulated by dietary levels of n-6 and n-3 PUFA. Rodents or piglets fed diets rich in the EC substrate AA showed greater 2-AG and AEA levels in the brain (Berger et al., 2001), small intestine, and liver (Artmann et al., 2008). Diets enriched with long chain n-3 PUFA (EPA and DHA) decreased AA levels and resulted in lower EC levels in the brain (Berger et al., 2001; Watanabe et al., 2003; Artmann et al., 2008; Wood et al., 2010), small intestine, liver (Artmann et al., 2008), visceral adipose tissue (Batetta et al., 2009), and plasma (Wood et al., 2010). These results demonstrate the link between dietary intake of PUFA, tissue PUFA concentrations, and EC levels.

As described, EC are products of dietary lipids. Modification of dietary fat intake can modulate the EC levels, both EPA and DHA can displace AA in cell membranes and then the derived EC, consequently reducing AEA and 2-AG production (Naughton et al., 2013). Similarly, oleoyl ethanolamide, a product of oleic acid, induces satiety, decreases circulating fatty acid concentrations, increases the capacity for β-oxidation, and inhibits the action of AEA and 2-AG in adipose tissue. The dietary lipids typically higher in n-6 PUFA drive the formation of AEA and 2-AG and likely support excessive energy intake and weight gain. Thus, understanding how dietary fats alter ECS activity is a pertinent area of research due to public health messages promoting a shift toward terrestrial plant and vegetable oils.

While dietary n-3 PUFA deficiencies have been linked to neuropsychiatric diseases (Parker et al., 2006), a potential underlying cause may be due to the desensitization and uncoupling of CB1 in the prelimbic prefrontal cortex and accumbens (Lafourcade et al., 2011). Recently, consumption of n-3 PUFA was shown to abolish negative consequences of non-functional CB1 activation regarding mood and behavior. In a study using 80 Male C57/blk6 mice (21-days-old), fed a modified AIN-93G diet (containing 11.04% fat) and assigned to either the control diet containing saﬄower oil or a DHA enriched diet (both diets were isocaloric and isonitrogenous), there were significant changes in the concentrations of n-3 and n-6 PUFA in tissues (serum, anterior tibialis, epididymal fat pads, and liver), including the brain (Kim et al., 2014b). After 62 days, mice fed the DHA diet had higher levels of 14:0, 16:1n7, 18:1n9, 18:2n6, 20:3n6, 22:5n3, and 22:6n3 in the brain, compared to the mice fed the control diet (**Table 1**; Kim et al., 2014b). Not surprisingly, the ratio of n-6/n-3 PUFA in the brain was significantly reduced in the DHA diet group, as compared to the control group (0.71 vs. 1.26). More specifically, the ratio of AA to DHA was reduced in the DHA diet fed mice (0.48 vs. 0.77). As shown in **Table 2**, after 118 days, brains from the mice given the DHA diet were observed to have higher levels of 14:0, 18:1n9, 18:2n6, 20:3n6, 20:5n3, 22:5n3, and 22:6n3, compared to the control diet fed mice (Kim et al., 2014b). Once again, the ratio of n-6/n-3 PUFA in the brain was significantly reduced in the mice fed the DHA diet, as compared to the mice fed the control diet (0.71 vs. 1.50). The ratio of AA to DHA was also reduced (0.48 vs. 0.86). The change in PUFA levels of the brain of mice given the DHA enriched diet were nearly identical to the levels found after 62 days. These data support the premise that dietary PUFA alter the fatty acid composition of the brain and the substrate for the biosynthesis of EC (Wood et al., 2010; McPartland et al., 2014). Dietary supplementation with n-3 PUFA predictably increased the concentration of EPA and/or DHA in tissues, cells, and plasma, and it decreased the relative concentration of AA. However, the data showing the effects of feeding n-3 PUFA to mice are more complex than a simple decrease in AA and increase in n-3 PUFA, as indicated by other aspects of their biochemistry.

**Table 1:** Mouse brain fatty acid composition after 62 days of feeding a semi-purified diet.

FA	Control		DHA		*t*-test *p* value
	Mean	SD	Mean	SD	
12:0	ND		ND		
14:0	0.13	0.00	0.14	0.005	0.012
14:1n5	ND		ND		
15:0	ND		ND		
16:0	19.38	0.13	19.13	0.22	0.0076
16:1t	0.15	0.004	0.13	0.002	<0.0001
16:1n7	0.44	0.02	0.47	0.01	0.0017
17:0	0.14	0.01	0.14	0.01	0.77
18:0	19.57	0.13	19.51	0.10	0.31
18:1n9	15.14	0.12	16.06	0.21	<0.0001
18:1n7	3.79	0.07	3.46	0.05	<0.0001
18:2n6	0.79	0.08	1.20	0.11	<0.0001
18:3n6	ND		ND		
18:3n3	ND		ND		
20:0	0.35	0.01	0.34	0.02	0.067
20:1n9	1.88	0.08	1.88	0.12	0.98
20:2n6	0.23	0.03	0.23	0.02	0.94
20:3n6	0.36	0.01	0.78	0.02	<0.0001
20:4n6	9.92	0.10	8.00	0.11	<0.0001
20:5n3	ND		0.02	0.04	0.17
22:0	0.21	0.01	0.22	0.01	0.35
22:1n9	0.19	0.01	0.18	0.01	0.55
22:4n6	2.99	0.06	1.89	0.04	<0.0001
22:5n6	2.06	0.09	ND		<0.0001
22:5n3	ND		0.19	0.01	<0.0001
22:6n3	12.96	0.17	16.71	0.25	<0.0001
24:0	0.25	0.02	0.26	0.02	0.16
24:1n9	0.31	0.06	0.33	0.05	0.60
TOTS	40.04	0.15	39.74	0.23	0.0047
TOTM	21.76	0.26	22.38	0.38	0.0009
PUFA	29.32	0.27	29.01	0.29	0.036
TN6	16.35	0.18	12.09	0.15	<0.0001
TN3	12.96	0.17	16.92	0.25	<0.0001
AA/DHA	0.77	0.01	0.48	0.01	<0.0001
n-6/n-3	1.26	0.02	0.71	0.01	<0.0001
Area%	91.27	0.13	91.27	0.11	0.95
Total area	4644	483	4397	397	0.25

*Values are weight percentages determined by gas chromatography (Watkins et al., 2010). *N* = 9 control group, and *n* = 9 for the DHA group. ND, not detected at the integration condition applied on these data.*

**Table 2:** Mouse brain fatty acid composition after 118 days of feeding a semi-purified diet.

FA	Control		DHA		*t*-test *p* value
	Mean	SD	Mean	SD	
12:0	ND		ND		
14:0	0.12	0.003	0.13	0.003	0.017
14:1n5	ND		ND		
15:0	ND		ND		
16:0	18.87	0.28	18.90	0.19	0.76
16:1t	0.15	0.005	0.13	0.01	<0.0001
16:1n7	0.47	0.02	0.48	0.01	0.093
17:0	0.13	0.01	0.13	0.003	0.73
18:0	20.09	0.14	19.93	0.07	0.0072
18:1n9	15.51	0.21	16.52	0.17	<0.0001
18:1n7	3.83	0.13	3.45	0.02	<0.0001
18:2n6	0.75	0.08	0.95	0.07	<0.0001
18:3n6	ND		ND		
18:3n3	ND		ND		
20:0	0.34	0.03	0.31	0.01	0.024
20:1n9	2.06	0.16	2.06	0.10	0.91
20:2n6	0.19	0.01	0.20	0.02	0.74
20:3n6	0.31	0.02	0.75	0.03	<0.0001
20:4n6	9.86	0.15	7.87	0.22	<0.0001
20:5n3	ND		0.09	0.03	<0.0001
22:0	0.21	0.02	0.20	0.01	0.18
22:1n9	0.19	0.01	0.18	0.01	0.032
22:4n6	3.25	0.09	1.99	0.04	<0.0001
22:5n6	2.90	0.19	ND		<0.0001
22:5n3	ND		0.20	0.01	<0.0001
22:6n3	11.51	0.24	16.29	0.24	<0.0001
24:0	0.24	0.03	0.23	0.02	0.44
24:1n9	0.40	0.05	0.36	0.06	0.20
TOTS	40.00	0.24	39.82	0.16	0.084
TOTM	22.46	0.39	23.05	0.29	0.0020
PUFA	28.77	0.39	28.33	0.32	0.017
TN6	17.26	0.26	11.75	0.18	<0.0001
TN3	11.51	0.24	16.58	0.26	<0.0001
AA/DHA	0.86	0.02	0.48	0.01	<0.0001
n-6/n-3	1.50	0.04	0.71	0.01	<0.0001
Area%	91.38	0.25	91.33	0.09	0.60
Total area	4646	256	4472	225	0.14

*Values are weight percentages determined by gas chromatography (Watkins et al., 2010). *N* = 9 control group, and *n* = 9 for the DHA group. ND, not detected at the integration condition applied on these data.*

## MUSCLE, LIVER, ADIPOSE AND THE ENDOCANNABINOID SYSTEM

The primary organs of macronutrient metabolism and energy expenditure that support growth and impact obesity and diabetes include the gastrointestinal tract, muscle, liver, adipose, and the endocrine system. The gastrointestinal tract is a site for the production of EC, and along with its associated organs, it is influenced by the ECS during the digestion and absorption of macronutrients. Muscle and liver are sites for glucose storage, as glycogen and adipose are for fat deposition. Furthermore, biochemical pathways of glucose, amino acids, and lipid metabolism link the muscle, liver, and adipose to cooperatively sustain the supply of intermediates that maintain energy balance in the fed and fasting state, as well as for growth and maintenance. It is clear that the ECS is a key player in macronutrient metabolism between the organs involved in food intake and systemic energy balance (Di Marzo and Matias, 2005; Viveros et al., 2008; Tibirica, 2010; Kim et al., 2013). At the gene level, sensitivity of muscle to insulin and glucose uptake appears to be influenced by the ECS (Kim et al., 2014a). Aspects of the involvement of the ECS in obesity, with specific studies in adipocyte cell cultures and adipose tissues, are described in the literature (Naughton et al., 2013). In many studies, the levels of EC and the activities of enzymes of EC synthesis and degradation suggest that a diet emphasizing n-6 PUFA, specifically AA, is a factor of concern.

We reported that feeding DHA to mice compared to feeding a semi-purified control diet resulted in an increase in the gene expression of cannabinoid receptors and enzymes for the synthesis/degradation of EC and for glucose metabolism (Kim et al., 2014b). The change in expression of ECS genes also indicated differences between muscle and adipose in mice. Furthermore, epididymal fat mass was lower in mice fed the DHA containing semi-purified diet, as compared to mice fed the control diet. If the action of DHA is as the EC, docosahexaenoyl ethanolamide, it may alter cannabinoid downstream signaling to improve glucose uptake in myoblasts (Kim et al., 2014a) and *in vivo*, the potential for n-3 PUFA to reduce diabetes is worthy of investigation. In support of these findings and the link to macronutrient metabolism, these initial studies in mice show that DHA, most likely through the ECS, alters metabolite profiles to favor fatty acid oxidation and reduce fat accretion (Kim et al., 2014b). Although these are early findings, they support the premise that dietary PUFA have roles in the ECS and eating behavior. At the least, mechanistic research in rodent models and clinical investigations on dietary PUFA in glucose metabolism and fat accretion are warranted to understand the relationships of fat intake and the ECS.

High circulating levels of AA-derived EC and excessive endocannabinoid production by adipocytes are associated with human obesity and fat accretion in rodents (Engeli et al., 2005; Cote et al., 2007). The ECS works through many anorexigenic and orexigenic pathways where ghrelin, leptin, adiponectin, endogenous opioids, and corticotropin-releasing hormones are involved (Viveros et al., 2008). Taken together, with the emerging role played by the ECS in obesity and with over production of the AA-derived EC prolonging stimulation of CB1 that leads to dysregulation (Matias et al., 2006, 2008), there is convincing evidence to focus future research on dietary PUFA and the ECS in metabolic syndrome.

Systemic macronutrient metabolism of carbohydrates, amino acids, and fatty acids now places the ECS as a target to redirect the fate of energy metabolism in the gastrointestinal tract, liver, muscle, and adipose. The ECS is an established player in CNS control of food intake. Emerging evidence of the phylogenic and developmental aspects of the ECS can be useful in understanding this complex system (Viveros et al., 2008). This research suggests that the genes for endocannabinoid enzymes, especially DAGL-α and NAPE-PLD, may contain alleles that express disease-related phenotypes, and therefore, place a greater emphasis on fully exploring the nature of how dietary PUFA influence activation of the cannabinoid receptors and downstream signals.

## CONCLUSIONS: ENDOCANNABINOIDS, EATING BEHAVIOR, AND AGING

As described in this review, the ECS plays an important role in eating, and specifically, when activated, CB1 leads to stimulation of food intake, which includes the behavioral aspects observed in fasted mice (Soria-Gomez et al., 2014). The mechanisms and actions of the ECS in the full array of the drive to eat are not well understood. Mechoulam and Parker (2013) recently reviewed several behavioral aspects of the ECS in the brain, suggesting that the brain contains numerous EC-like compounds that activate the cannabinoid receptors to influence mood, depression, cognition, and learning; however, further study is needed to advance the knowledge of the CNS and brain functions. Interestingly, the distribution of the CB1 receptors, which is a primary receptor in the brain (Wilson and Nicoll, 2002) and CNS, differs in neonatal and adult brains. Moreover, since cannabinoid receptor distribution and expression are influenced by aging, research should determine how the ECS functions during aging and especially with the loss of appetite in older adults. These findings underscore the complex nature of the ECS, which is a comprehensive component that ultimately impacts all aspects of eating behavior.

It is well known that the ECS and the endogenously produced EC are crucial components for inducing food intake and controlling macronutrient metabolism (Di Marzo and Matias, 2005). The flux through metabolic pathways for glucose, fatty acids, and amino acids is largely dependent on rate regulating enzymes of metabolic pathways in major organs, such as muscle, liver, and adipose, which are all impacted by the ECS. Hence, understanding these intermediary pathways, endocrine factors, and gene expression will encourage important areas of research to determine the role of the ECS on eating behavior (Richard et al., 2009). As previously stated, one research approach should be to characterize how the different PUFA families integrate in the processes of metabolic flux of macronutrient pathways in major organs where the ECS is of consequence.

In this review, we described the current research on the ECS and food intake, macronutrient metabolism, and the participating dietary PUFA that serve as substrate for the biosynthesis of EC. The evidence clearly indicates that the activation of the cannabinoid receptors in the brain stimulates food intake and overstimulation of the ECS contributes to overeating and obesity. The newly discovered feature of the ECS is that diet, specifically PUFA, influences genes of the ECS in cells and tissues. In this regard, the ECS participates in macronutrient metabolism and energy status in adipose and muscle that appears to be highly dependent on the type of EC derived from dietary PUFA. Future studies should focus on the underlying aspects of how the n-6 and n-3 dietary PUFA families affect the ECS, alter the types of EC synthesized, change gene expression of the ECS, and direct the signaling pathways downstream of receptor activation. These investigations will potentially impact obesity risk and metabolic syndrome.

## Conflict of Interest Statement

The authors declare that the research was conducted in the absence of any commercial or financial relationships that could be construed as a potential conflict of interest.

## Abbreviations

AA: arachidonic acid
AEA: arachidonylethanolamide
2-AG: 2- arachidonoylglycerol
DHA: docosahexaenoic acid
EC: endocannabinoids
ECS: endocannabinoid system
EPA: eicosapentaenoic acid
FAAH: fatty acid amide hydrolase
MAGL: monoacylglycerol lipase
NAE: *N*-acylethanolamine
NAPE-PLD: p *N*-acyl phosphatidylethanolamine selective phospholipase D
PUFA: polyunsaturated fatty acids.
